# Effectiveness and tolerability of rectal ointment and suppositories containing sucralfate for hemorrhoidal symptoms: a prospective, observational study

**DOI:** 10.1007/s00384-024-04642-7

**Published:** 2024-05-16

**Authors:** Anikó Rita Marik, Ildikó Miklós, Gábor Csukly, Péter Hársfalvi, András Novák

**Affiliations:** 1 Egis Pharmaceuticals PLC, Budapest, Hungary; 2 Semmelweis University, Budapest, Hungary; 3 Bitrial Clinical Research Kft, Budapest, Hungary

**Keywords:** Hemorrhoidal disease, Sucralfate ointment, Sucralfate suppository, Symptoms, Topical medical device

## Abstract

**Background and aims:**

A high number of topical products are available for the treatment of hemorrhoidal symptoms. Sucralfate-based topical products constitute a new treatment alternative that act as a mechanical barrier to facilitate healing. The aim of this prospective, observational study was to determine patient- and physician-assessed effectiveness and tolerability of rectal ointment and suppositories containing sucralfate for the treatment of hemorrhoidal symptoms in routine clinical practice.

**Methods:**

Adult patients with diagnosed, mild-to-moderate, symptomatic non-bleeding hemorrhoids treated with rectal ointment or suppositories containing sucralfate were enrolled. Patients were administered treatment twice per day for at least 1 week until symptom resolution and/or for a maximum of 4 weeks. The primary endpoint was patient-assessed effectiveness on a modified Symptom Severity Score (mSSS, range 0 to 14). Physician-assessed effectiveness (9 symptoms, 0 to 5 Likert scale), hemorrhoid grade, and patient satisfaction were also determined.

**Results:**

Five investigators enrolled 60 patients; mean age was 48.4 ± 16.6 years and 72.4% were female. Pain or pressure sensitivity was reported as the most severe symptom by patients, and pressure sensitivity, discharge, soiling, and prolapse by physicians. Mean patient-assessed mSSS at baseline was 6.6 ± 1.9 and was significantly improved overall and in the ointment and suppository groups individually by −4.6 ± 2.0, −4.4 ± 1.8, and −4.8 ± 2.2, respectively (*p* < 0.0001). Investigator-assessed mean baseline symptom score was 18.1 ± 3.9 and improved by −7.1 ± 4.5, −6.9 ± 5.4, and −7.3 ± 3.5, respectively (*p* < 0.0001). Investigator-assessed symptoms of pressure sensitivity, swelling, and discharge were improved to the greatest extent. Hemorrhoid grade was improved in 38% of patients at the end of treatment. Compliance with treatment was 97.4% and patient satisfaction with application and onset of action was high (81.3% and 76.2%, respectively). Both the ointment and suppository were well tolerated.

**Conclusions:**

The effectiveness of topical ointment or suppository containing sucralfate on patient- and investigator-assessed hemorrhoidal symptoms in real-life clinical practice was demonstrated. Patient satisfaction was high and treatments were well tolerated. Larger controlled trials are warranted to confirm the results.

## Introduction

Hemorrhoidal disease is one of the most common proctologic diseases. It has been estimated that up to 50% of the population will experience symptomatic hemorrhoidal disease at some point in their lives [1, 2], but the reported prevalence is highly variable, ranging between 11 and 12.8% [3, 4] and 39% [5] of the adult population, and reaching 50% or more in certain age groups [5, 6]. In both sexes, the peak incidence occurs between the ages of 45 and 65 years [1, 4]. Factors contributing to a high prevalence include poor diet and bowel habits, such as inadequate fiber intake, straining at defecation and prolonged sitting on the lavatory, as well as constipation or diarrhea. Conditions associated with elevated intraabdominal pressure such as pregnancy, overweight, obesity, ascites, and pelvic space-occupying lesions can also lead to the development of hemorrhoids [1, 7–9]. Common clinical features associated with the condition are anal pain or pressure sensitivity, rectal bleeding, discomfort, itching, swelling, soiling and fecal incontinence, pruritus, and prolapse [3, 7, 9–11].

Hemorrhoids develop when the supporting collagen fibers and fibroelastic tissues of the anal cushions deteriorate causing an abnormal downward displacement of the cushions and venous dilatation, with subsequent prolapse, bleeding, and thrombosis [9]. Although hemorrhoidal disease may resolve spontaneously or with conservative medical therapy, the associated symptoms may be severe and disabling for patients and can cause significant worsening of the patient’s quality of life [12]. Untreated hemorrhoids can lead to complications such as strangulation, incarceration, severe bleeding, thrombosis, secondary infection, ulceration, and incontinence [11, 13]. Moreover, symptoms often reappear, with an estimated 5-year recurrence rate of 10–50% with nonsurgical techniques, and less than 5% with surgical hemorrhoidectomy [13].

Topical preparations are typically used for initial treatment of hemorrhoids with the aim of controlling or relieving symptoms [9, 14–16]. This coincides with patient preference as they seek easily available solutions, and mostly initiate treatment without consulting a physician [3] because of embarrassment or fear resulting from the nature and location of the condition. A range of over-the-counter products are available for the treatment of symptomatic hemorrhoids, often with little or no scientific evidence to support their use [17]. Given the prevalence of the disease, its disabling effects, and its intimate character, there remains an unmet need for effective, evidence-based initial treatments.

A new topical product (ointment and suppository) has been developed to fill this treatment gap and to provide patients with an alternative to cortisone- or lidocaine-based treatments. It has a medical device status due to its physical action. The product contains sucralfate (3% for the rectal ointment and 2% for the suppository), a negatively charged complex of sucrose, and aluminum hydroxide, which binds to positively charged proteins [18] present on the anal mucosa lesion, leading to the formation of a mechanical barrier. The product also contains low concentrations of herbal extracts including calendula, witch hazel leaf (hamamelis), and chamomile, which act as skin conditioning and soothing agents. This new topical product covers and protects the epidermis; soothes the inflamed, itchy skin; and by maintaining a suitable environment helps to promote skin regeneration and diminish skin dehydration, thereby aiding wound healing, and reducing the risk of fissure and injury during defecation.

The current study was conducted to provide evidence for the effectiveness of topical treatments containing sucralfate in real-life practice. Both the sucralfate-containing rectal ointment and suppository have been on the market since 2017, without any data to suggest insufficient performance or lack of safety with long-term use. For these reasons, an open-label, prospective, observational study without comparators was chosen in which both patients and medical specialists evaluated the performance of the medical devices under everyday conditions.

## Materials and methods

This prospective, observational, longitudinal, uncontrolled, multi-center study was conducted between April and August 2021 at five study centers in Hungary by four surgeon-proctologists and one general practitioner.

The aim of the study was to collect real-life data on the effectiveness and safety of topical medical device products containing sucralfate (Reparon^®^ Herbal rectal ointment and Reparon^®^ Herbal rectal suppository, Egis Pharmaceuticals PLC, Budapest, Hungary). As the two forms were assumed to be identical in terms of effectiveness and safety, both products were assessed in the same study.

The study recruited male and female subjects consecutively with a diagnosis of hemorrhoidal disease prescribed a sucralfate-containing ointment or suppository by the investigators as part of routine clinical practice. The choice of treatment was independent of the study and based on the investigator’s judgement and usual clinical practice. The maximum treatment duration followed the Instructions for Use of these topical products and was similar to previously published studies with other sucralfate-containing products [19] or with similar ointments and suppositories [20–22] used in the same indications. Individuals ≥ 18 years of age with non-bleeding hemorrhoids were enrolled. Patients were not eligible for the study if they met any of the following criteria: suspected hypersensitivity and/or a contraindication to any of the product ingredients; bleeding hemorrhoids in the previous 2 weeks; anal surgery and/or other inflammatory anal diseases in the previous 30 days; planned anal surgery during the study period; lack of compliance to study treatment; participation in other studies in the 30 days before recruitment; and presence of other significant disease(s) as determined by the investigator.

At the enrollment visit (V1), all patients who had been prescribed treatment with either the sucralfate-containing ointment or suppository received the products. Patients used the products rectally twice per day after a bath or shower (as directed by the treating physician and the product Instructions for Use) for at least 1 week and until a symptom- and/or a complaint-free condition was reached, and for a maximum of 4 weeks. At this point, the end of study visit (V2) took place. The maximum study period was based on the recommended treatment period for the products.

At visits V1 and V2, enrolled patients underwent a clinical examination which could include anoscopy, rectoscopy, rectal ultrasonography, microbiology, or cytology as deemed necessary by the investigator. As the treatments are typically used without medical supervision in routine clinical practice due to the intimate nature of the disease, the emphasis when evaluating effectiveness was placed on patient perception. For the primary endpoint, patients self-rated their symptoms on a modified version of a previously published Symptom Severity Score (mSSS) [23]. Using this scale, symptoms of itching/discomfort, pain/pressure sensitivity, and soiling were scored from 0 (none/never) to 3 (persistent/constant/incontinent), and patient-perceived disease severity was scored from 1 (no trouble) to 5 (really bad) giving a minimum score of 1 and a maximum score of 14 (Table 1). The original symptom severity score was developed to assess symptom severity following anopexy or hemorrhoidectomy [23]. For the purposes of the current study, the score was therefore adjusted to more accurately reflect the change in symptoms expected with topical therapy. Alleviation of bleeding, prolapse, and gas incontinence are not the intended use of sucralfate-based therapy, and scoring for these symptoms was therefore removed. Bleeding was assessed separately as part of the safety evaluation if it was present.

**Table 1:** Modified symptom severity score used in the study

**Symptom severity score**							
Score	Itching/discomfort			Pain/pressure sensitivity		Soiling	
0	Never			None		Never	
1	Occasional			With stool		Mucus discharge	
2	Regular			With stool and after		Occasional	
3	Persistent			Constant (independent of stool)		Incontinent	

**Disease severity score (How troublesome are your piles? )**							
No trouble		Mild	Moderate		Severe		Really bad
1		2	3		4		5

As secondary endpoints, objective assessments were provided by the investigators who determined the effectiveness of therapy on symptoms of erythema, edema, rhagades, and fissure ani, wounds, pressure sensitivity, dryness of anal skin, anal discharge/moisture, swelling of nodes, and degree of prolapse of internal hemorrhoids on a 5-point Likert scale (1 = not present, 5 = worst severity, minimum score = 9, maximum score = 45). Hemorrhoid grading (grades I–IV) was assessed according to Table 2. This assessment was based on Goligher’s classification [3, 9, 15, 24]. This defines hemorrhoid grades based on morphological differences alone according to degree of prolapse and does not include the various subjective symptoms associated with diverse hemorrhoids [25]. Nevertheless, it is an excellent endpoint as internal hemorrhoids can be categorized without the need for an instrumental examination and only on the basis of the degree of prolapse. Other hemorrhoidal symptoms were assessed with different objective and subjective endpoints. Moreover, the anal region was photographed before and after the treatment and the physicians evaluated the overall status change (worsened/no change/improved) based on perceived differences in skin redness, swelling, prolapse, signs of bleeding, other visible discharge, signs of excoriation and rhagades, and other sores or fissures.

**Table 2:** Hemorrhoid grading based on Goligher’s classification as used in the study

Grade I hemorrhoids	May bleed but do not prolapse; on colonoscopy, they are seen as small bulges into the lumen
Grade II hemorrhoids	Prolapse outside the anal canal but reduce spontaneously
Grade III hemorrhoids	Protrude outside the anal canal and usually require manual reduction
Grade IV hemorrhoids	Are irreducible and constantly prolapsed. Acutely thrombosed hemorrhoids and those involving rectal mucosal prolapse are also grade IV

At V2, patients’ compliance (adherence to the treatment) and analgesic use during the study were recorded, as well as the number of days until symptom alleviation, and patients’ satisfaction with the products (very satisfied/satisfied/unsatisfied/very unsatisfied) in terms of application and speed of onset of action.

Safety was assessed in terms of adverse events and device deficiencies, including bleeding accidents and incidents leading to study withdrawal. Physical examination findings and vital sign measurements were performed in accordance with routine clinical practice.

The use of all prior and concomitant medication necessary for the welfare of the patients was at the discretion of the physicians and was recorded. According to the study protocol, patients were excluded if they used other treatments for hemorrhoids (including local or oral treatments) or wet wipes.

Data for the primary and secondary endpoints, subject demographic characteristics, and medical history were recorded at the time of enrollment (V1) and at the V2 follow-up visit after approximately 2–4 weeks and collected in an electronic case report form (eCRF). In between study visits, patients’ usage data, daily mSSS, and use of analgesics were recorded in patient diaries.

This paper adhered to the STROBE guidelines for the reporting of observational studies. The study complied with the ethical principles derived from the revised Declaration of Helsinki (2013). The study protocol was approved by the Committee of Science and Research of the Medical Research Council and received approval from the National Institute of Pharmacy and Nutrition (OGYÉI) in Hungary. All patients provided written informed consent.

### Statistical analysis

Regarding sample size, the aim was to achieve a precision estimate of at least 0.6 points in terms of the halfwidth of the 95% confidence interval (CI). As the effect size was unknown and no previous studies with the products were available, the mean of the baseline standard deviations was used [26] from a study of a different ointment (containing sucralfate) with similar indications [20]. Thus, under the assumption that the standard deviation of the change in mSSS is 1.5 points, and the precision estimate in terms of the 95% CI is ± 0.6 points, a minimum of 24 patients had to be included in the analysis for both products. In order to ensure this sample size with an expected 20% drop-out rate, an inclusion rate of 30 patients per product was targeted.

The modified intention-to-treat (mITT) set comprised all participants who were treated at least once and had primary effectiveness assessments at baseline and at least one post-baseline value. The last observation carried forward (LOCF) approach using the mSSS from the patient diary was used for imputing missing values as it is associated with the least bias in analyses without a comparator arm [27, 28].

The per protocol population (PPP) consisted of all participants who had a baseline and end-of-study effectiveness measurement, and whose treatment compliance was between 70 and 120%. Analysis of the primary effectiveness endpoints was performed on both the mITT and PPP.

All subjects who treated themselves at least once were included in the safety analysis set (SAS). Summary statistical analyses were provided for all incidents collected.

The analysis of the outcome variables was conducted using the following descriptive statistics. For continuous variables: the number of observations and missing values, and the mean, standard deviation, median, p25 (Q1), p75 (Q3), minimum, and maximum. For nominal and ordinal variables: the number of observations and missing values, frequencies, and percentages for each category of the variable (excluding the missing data from the denominator).

Two-sided 95% confidence intervals (CI) were estimated for the changes between V2 (study end) and V1 (baseline).

Within-group analyses were performed using a paired *t*-test for each galenic form, with *p* < 0.05 considered significant. No between-group comparisons were performed.

The data were recorded electronically and statistical analyses were carried out at BiTrial using R Statistical Software (v4.1.2; R Core Team 2021).

## Results

### Demographic and baseline characteristics

A total of 60 patients met the inclusion criteria and were enrolled by the five investigators. The mITT group consisted of 58 patients (29 in each group) as two patients were withdrawn during the study: one was excluded from the suppository group due to bleeding, and one subject in the ointment group discontinued all study activities and communication. The LOCF method was applied for three patients. All 60 patients received at least one dose of treatment and were included in the safety set. In the PPP set, 48 patients (24 in each group) were analyzed. The results of the mITT and PPP analyses were very similar, and only the mITT results are presented here.

Subject demographic and baseline characteristics are shown in Table 3. The mean age was 48.4 ± 16.6 years and 72.4% of subjects were female. There was a slightly higher proportion of women in the ointment than in the suppository group (75.9% vs. 69.0%, respectively), and hemorrhoid duration was longer in the suppository group than the ointment group (40.8 ± 54.3 vs. 31.0 ± 68.0 months, respectively).

**Table 3:** Patient demographics and baseline characteristics

	Suppository group (mITT, *N* = 29)		Ointment group (mITT, *N* = 29)		Total mITT (*N* = 58)
Sex - female, *n* (%)	20 (69.0%)		22 (75.9%)		42 (72.4%)
	Min, max	Mean ± SD	Min, max	Mean ± SD	Mean ± SD
Age (years)	22, 75	49.0 ± 14.7	21, 83	47.8 ± 18.5	48.4 ± 16.6
Illness duration^a^ (months)	0, 187	40.8 ± 54.3	0, 358	31.0 ± 68.0	35.9 ± 61.2

**Modified Symptom Severity Score – patient’s perceived symptom severity**					
Total mSSS scale (scale 1–14)	2, 11	6.7 ± 2.2	3, 9	6.5 ± 1.5	6.6 ± 1.9
Itching/discomfort (scale 0–3)	0, 3	1.4 ± 0.6	1, 2	1.3 ± 0.5	1.4 ± 0.6
Pain/pressure sensitivity (scale 0–3)	0, 3	1.6 ± 0.8	0, 3	1.7 ± 0.6	1.6 ± 0.7
Soiling (scale 0–3)	0, 2	1.0 ± 0.8	0, 2	1.0 ± 0.7	1.0 ± 0.8
Disease severity (scale 1–5)	1, 4	2.7 ± 0.6	2, 3	2.5 ± 0.5	2.6 ± 0.6

**Symptoms assessed by investigators**					
Total symptom load (scale 9–45)	11, 26	18.1 ± 4.0	12, 31	18.1 ± 3.8	18.1 ± 3.9
Erythema (scale 1–5)	1, 3	1.6 ± 0.6	1, 3	1.9 ± 0.6	1.7 ± 0.7
Edema (scale 1–5)	1, 3	1.9 ± 0.6	1, 3	1.9 ± 0.7	1.9 ± 0.6
Rhagades and fissure ani (scale 1–5)	1, 3	1.4 ± 0.6	1, 3	1.6 ± 0.7	1.5 ± 0.6
Wounds (scale 1–5)	1, 4	1.4 ± 0.7	1, 3	1.5 ± 0.7	1.5 ± 0.7
Pressure sensitivity (scale 1–5)	2, 4	2.7 ± 0.7	2, 4	2.7 ± 0.6	2.7 ± 0.6
Dryness (scale 1–5)	1, 3	1.9 ± 0.9	1, 3	1.7 ± 0.8	1.8 ± 0.9
Discharge (scale 1–5)	1, 4	2.3 ± 0.9	1, 4	2.5 ± 0.8	2.4 ± 0.9
Swelling (scale 1–5)	1, 5	2.5 ± 1.0	1, 4	2.1 ± 0.9	2.3 ± 1.0
Prolapse (scale 1–5)	1, 4	2.4 ± 0.9	1, 4	2.2 ± 0.9	2.3 ± 0.9

^a^Time from the first diagnosis/observation of hemorrhoids

Subjects had mild-to-moderate disease severity with a baseline mSSS of 6.6 ± 1.9. Pain or pressure sensitivity was reported as the most severe symptom with an individual mSSS score (on a 0 to 3 scale) of 1.6 ± 0.7 (Table 3). Individual mSSS scores for itching/discomfort and soiling were 1.4 ± 0.6 and 1.0 ± 0.8, respectively. Subjects perceived their disease and its symptoms as moderate on the disease severity score (2.6 ± 0.6).

Investigator-assessed symptoms as total symptom load were also mild-to-moderate in severity with a mean baseline score of 18.1 ± 3.9 on the 9–45 Likert scale. The most severely rated symptoms were pressure sensitivity, discharge, soiling, and prolapse. Based on the hemorrhoid grading, just under two-thirds of patients presented with grade II symptoms (63.8%) and just under one-third with grade III symptoms (31.0%) (Table 4). Although there were no significant differences between the suppository and ointment user groups in regard to the above scores, the baseline symptoms of swelling, prolapse, and dryness were rated slightly more severe in the suppository group, while discharge, erythema, rhagades or fissure ani, and wound symptoms were rated more severe in the ointment group and slightly more grade III patients presented for this group.

**Table 4:** Hemorrhoid grades based on Goligher’s classification on visits 1 and 2 and the number of patients improving in the mITT group

Hemorrhoid grading	Number of patients *n*, (% of mITT group)		Number of patients improved at V2 *n*, (% of V1 grade group)	
	On V1 (*N* = 58)	On V2 (*N* = 55)	With 1 grade	With 2 grades
Grade I	2 (3.4%)	13 (23.6%)	10 (27.0%)	1 (5.6%)
Grade II	37 (63.8%)	36 (65.5%)	10 (55.6%)	0 (0%)
Grade III	18 (31.0%)	5 (9.1%)	0 (0%)	
Grade IV	1 (1.7%)	1 (1.8%)		

Besides hemorrhoids, 20 patients (34.5% of the mITT) had other concomitant diseases (such as hypertension, reflux disease, gout, or cardiovascular diseases) and 18 used medications for these conditions during the study. No clinically significant abnormality or alteration in the disease state or medication compared to baseline was detected at the follow-up visit.

### Duration of treatment and compliance

The mean duration of treatment was 19.1 ± 4.6 days with the suppository and 20.4 ± 5.1 days with the ointment, and the average number of applications was 1.9 ± 0.2/day. Compliance was high during the study with an average of 97.4% in the mITT. In addition, 80% (48 subjects) of all enrolled subjects had compliance of 70–120% defining the PPP group with 98.7% average compliance.

### Primary endpoint: patient-assessed symptoms

**Fig. 1:**
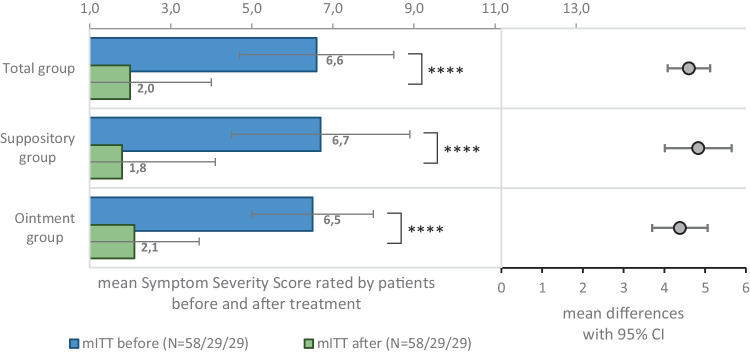
Modified symptom severity score (min = 1, max = 14) assessed by patients before and after treatment (mean scores) and 95% confidence intervals. ****p <0.0001 (paired *t*-tests)

**Fig. 2:**
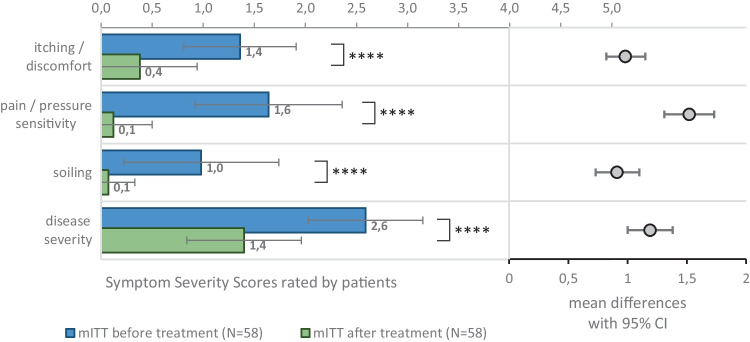
Symptoms assessed by patients on the modified symptom severity score before and after treatment (mean scores and 95% confidence intervals). ****p  < 0.0001 (paired *t*-tests)

Summed mSSS values at V1 and V2 are presented in Fig. 1, and the individual mSSS symptom scores in Fig. 2. The change of the summed mSSS from baseline at V2 was significant with a mean decrease of −4.6 ± 2.0 (*p* < 0.0001) in the whole mITT sample, and −4.8 ± 2.2 and −4.4 ± 1.8 in the suppository and ointment user groups, respectively (*p* < 0.0001). Moreover, the change in mSSS evaluated separately by symptoms showed significant improvement in each score in the mITT population. A forest plot illustrating the changes in mSSS is presented in Fig. 2.

### Investigator-assessed symptoms and clinical features

**Fig. 3:**
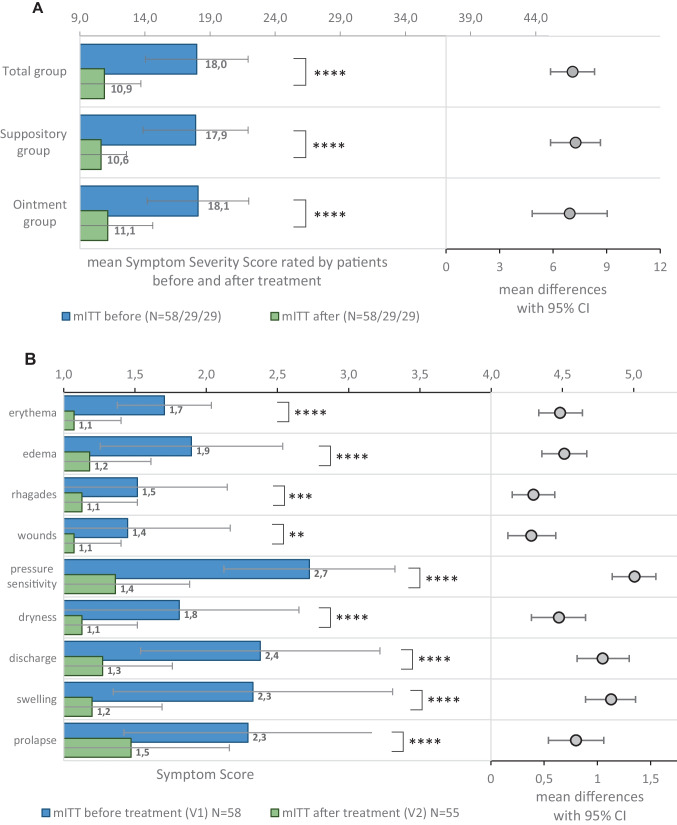
** A** Investigator-assessed cumulative symptom load (min = 9, max = 45) before and after treatment (mean scores) and 95% confidence intervals. ****p < 0.0001 (paired *t*-tests). **B** Investigator-assessed symptoms on Likert scale [1–5] before and after treatment (mean scores 95% confidence intervals). **p < 0.01, ***p < 0.001, ****p < 0.0001 (paired *t*-tests)

Patients showed a significant improvement at V2 relative to baseline in the investigator-assessed symptoms. The mean change in the cumulative symptom load was −7.1 ± 4.5 (*p* < 0.0001) for the whole mITT population, and −7.3 ± 3.5 (*p* < 0.0001) and −6.9 ± 5.4 (*p* < 0.0001) in the suppository and ointment groups, respectively. Of the nine investigator-assessed symptoms, pressure sensitivity, swelling, and discharge were improved to the greatest extent, with scores significantly decreased by 49.9%, 48.4%, and 46.5%, respectively, compared with baseline. Improvements were also observed for dryness, edema, erythema, and prolapse symptoms with a significant decrease of 37.7–35.8% compared with baseline. Symptom ratings before and after treatment as well as the difference between visits are presented in Fig. 3.

A significant improvement in hemorrhoid grading was observed at V2 relative to baseline. Specialists categorized 21 (38%) patients’ disease into a lower grading category (grade I or II) at the end of treatment visit (V2). This resulted in a mean decrease in the grading classification of −0.40 ± 0.5 (*p* ≤ 0.0001), −0.48 ± 0.6 (*p* = 0.0002), and −0.32 ± 0.5 (*p* = 0.0013) for the total, suppository, and ointment mITT groups, respectively. The number of patients in each grade and the number of patients with improvement are presented in Table 4. At V2, nearly a quarter of patients (23.6%) were classed as grade I, compared with only 3.4% at baseline (Table 4). Among patients in the total mITT group who experienced an improvement in hemorrhoid grade, more marked improvements were observed for the symptoms of prolapse and swelling (−0.86 and −0.41 Likert score difference, respectively), and lesser improvements in discharge and pressure sensitivity (−0.30 and −0.29 score difference, respectively) compared with patients whose hemorrhoids remained in the same grade.

**Fig. 4:**
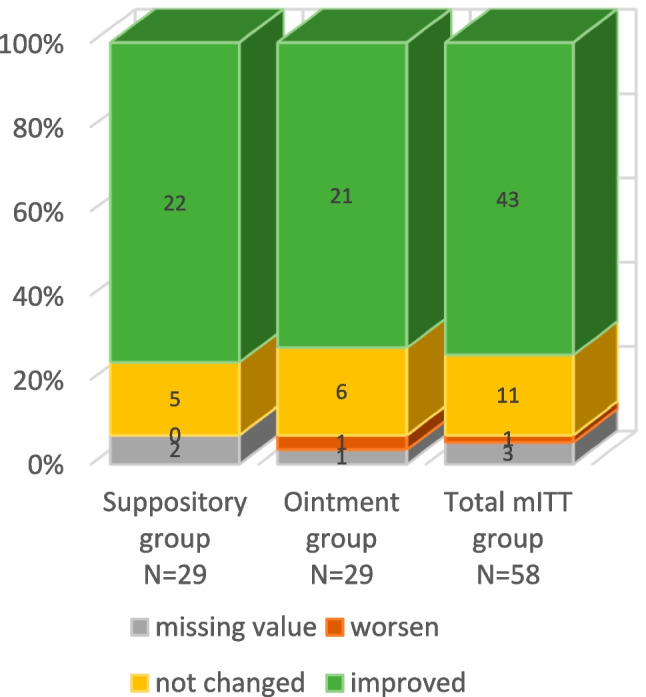
Investigators’ assessment of the change of disease based on photo documentation

**Fig. 5:**
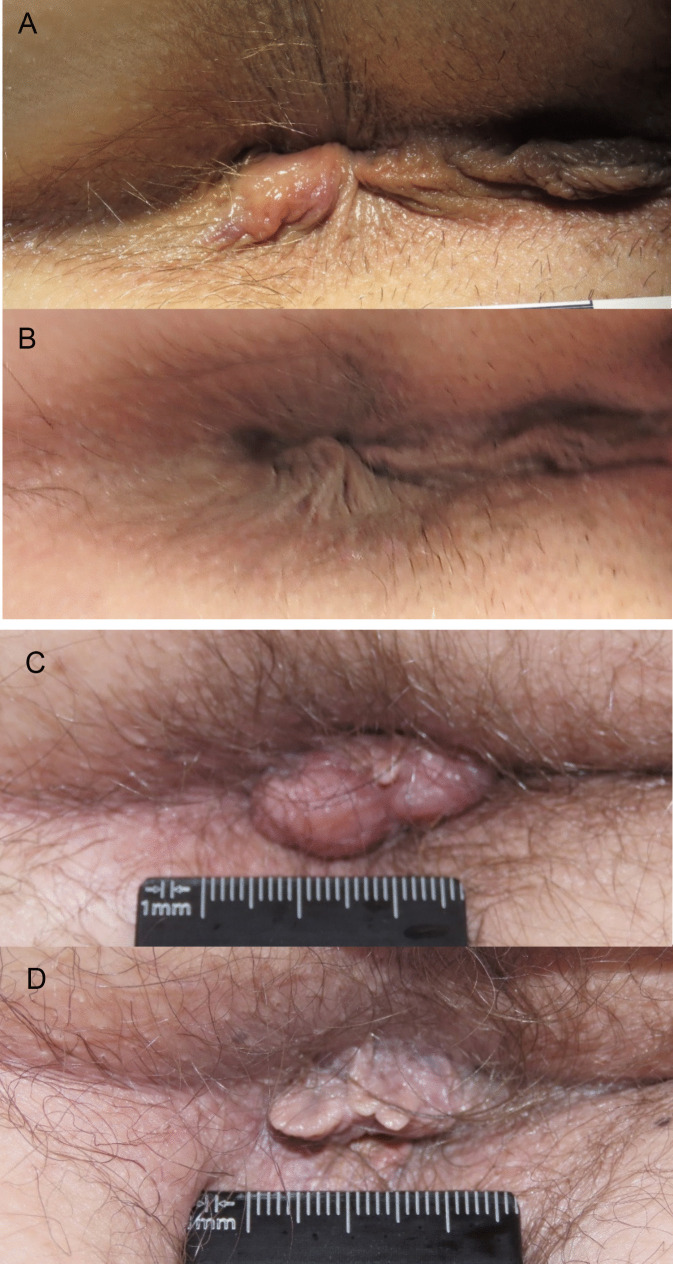
**A**–**D** Examples of 2 cases with improved overall change.** A **and **C** are captured before, **B** and **D** are after the treatment

Based on the photographic documentation, investigators rated the overall change in clinical features as “improved” in 74.1%, “not changed” in 19.0%, and “worsened” in 1.7% (5.2% had missing photographic data or dropped out of the mITT). Figure 4 presents the change based on photographic documentation across all groups and Fig. 5 is a photographic illustration of the improvements.

### Patient satisfaction

**Fig. 6:**
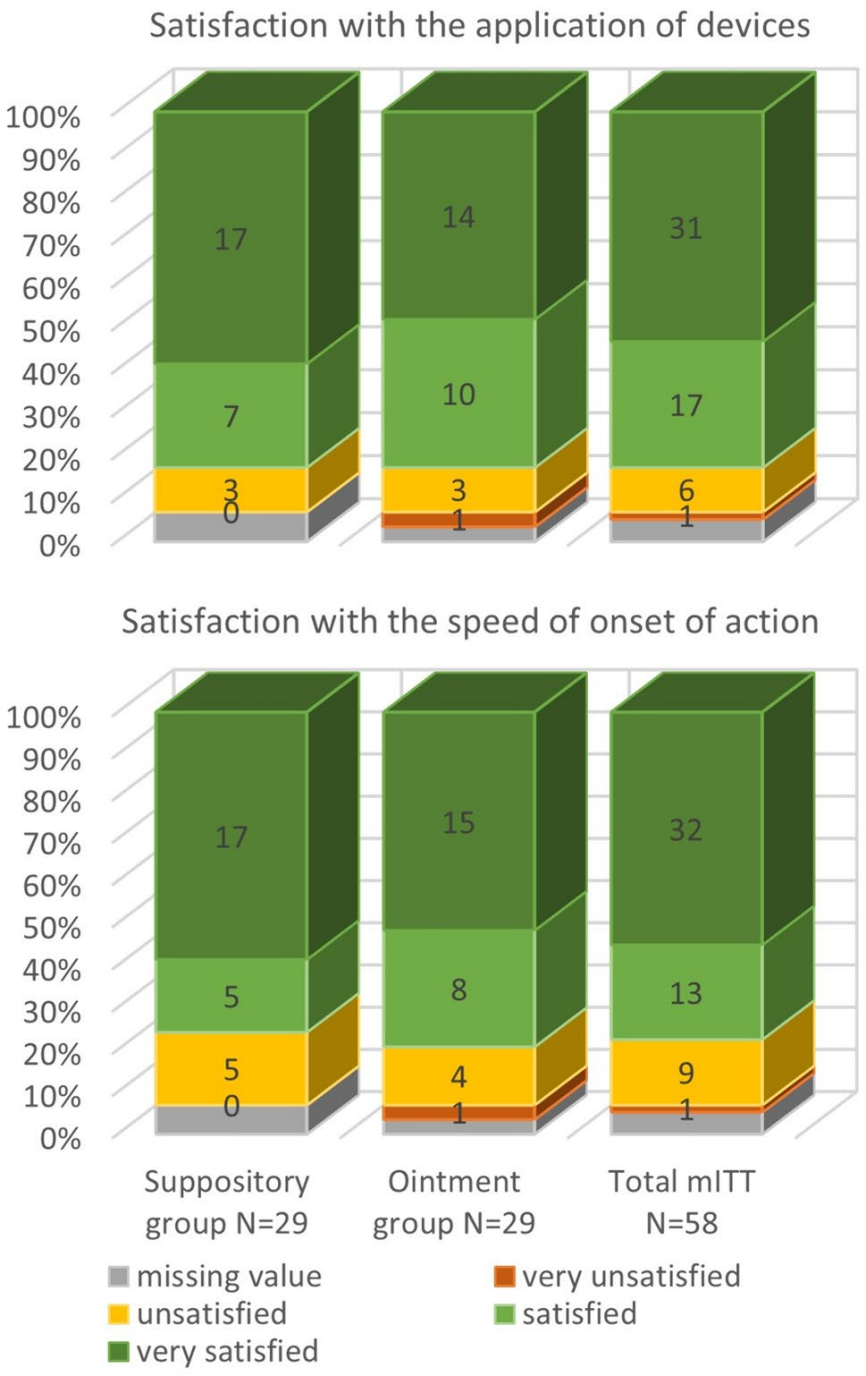
Patients’ satisfaction with the devices

The majority of the enrolled patients were satisfied with the products. Satisfaction with the application of the devices was reported by 81.3% of the total mITT population, 83.3% of the suppository group, and 80.0% of the ointment group. Satisfaction with the speed of onset of action was reported by 76.2% of the total mITT population, 76.7% of the suppository group, and 75.8% of the ointment group. The overall mean elapsed time to symptom alleviation was 7.9 ± 4.7 days, 8 ± 5.1 days, and 7.8 ± 4.3 days in the total, suppository, and ointment mITT groups, respectively. Figure 6 presents the patient satisfaction data in detail.

### Safety and tolerability

In this study, the medical devices were used a total of 2182 times. During the study period, one pregnancy was reported in a patient using the suppository twice daily for 20 days without any issues. One device-related safety event occurred in a patient using the suppository mostly twice daily for 28 days (using it altogether 59 times), when the suppository was rigid after removing it from the fridge and administration disturbed the rectal mucosa causing mild bleeding. In all other patients, both medical devices were well tolerated and safe in a real-life setting for patients with hemorrhoids. Although necessary concomitant medications were recorded for other coexisting diseases (at the discretion of the investigators), no patients used other hemorrhoidal treatments and only one patient used an analgesic during the study.

## Discussion

The findings of this real-life observational study — with significant reductions in hemorrhoidal symptom load and severity — provide compelling evidence supporting the effectiveness of topical medical device products containing sucralfate, both ointment and suppositories. Improvements were observed with both patient- and physician-assessed symptom severity scores. Treatment was particularly effective at relieving pain and pressure sensitivity, itching, and swelling, which are among the most common symptoms of hemorrhoidal disease [3, 7, 10, 12] and can significantly impair quality of life [29]. Investigators also noted a significant improvement in hemorrhoid grading at the end of treatment compared with baseline with 38% of patients improving by at least one grade. Patients whose hemorrhoid grade was reduced showed a greater improvement in symptoms, in particular prolapse and swelling, compared with those whose grade remained the same.

Sucralfate-based products were designed to promote healing by providing a physical barrier over the affected site, and have been widely used for the treatment of gastric and duodenal ulcers as well as epithelial wounds [30–33]. These barrier therapies work convincingly as they restore and protect the physical and functional integrity of the mucosal gel layer and maintain healthy homeostasis within the biology of the mucosal barrier, which is essential in the management of clinical syndromes characterized by physical disruption of the mucosa [34]. The rectal topical medical device products containing sucralfate provide relief from hemorrhoidal symptoms by coating the surface and forming an insoluble adherent complex with the mucosal proteins, a physical barrier that protects the site from mechanical damage and new lesions [34]. The addition of herbal extracts including calendula, chamomile and witch hazel, which have documented anti-inflammatory and wound healing properties in higher concentrations [35, 36] and skin conditioning and soothing effects in the applied concentrations, contributes to the treatment’s effectiveness. Hemorrhoidal specimens display an inflammatory reaction involving the vascular wall and surrounding connective tissue, with associated mucosal ulceration, ischemia, and thrombosis [37]. It is thought that application of the sucralfate-containing rectal products prevents tissue injury through covering and protecting the epidermis, and consequently diminishes the release of inflammatory cytokines from damaged epithelial cells, reducing inflammation and edema [31], thereby contributing to the improvement in swelling and prolapse reduction observed in the current study. A reduction in inflammation will also reduce mucus discharge, providing the skin with an opportunity to regenerate and heal, thereby supplementing the epidermal protection provided by the topical product itself. For the patient, this leads to a less painful and less pressure-sensitive condition, with fewer symptoms and smaller, more pliable prolapses, which are more likely to reduce spontaneously. A similar explanation has been put forward to describe the observed benefits of sucralfate-based treatment on post-hemorrhoidectomy wound healing and pain reduction [38].

Topical treatments, alone or in combination with oral venous active drugs [9, 15, 16], form one of the first-line, conservative treatments mainly for low-grade and non-thrombosed hemorrhoids, along with dietary and lifestyle modifications. The main goal of such treatment is to control the acute symptoms rather than to cure the underlying hemorrhoids. They are generally available without prescription and are popular among patients, especially as most do not seek medical advice when symptoms first occur [3]. Given that hemorrhoidal disease is a common affliction in the adult population [1–6] and can cause distressing symptoms even at a low grade, it is important that available treatments are supported by clinical effectiveness data. This post-marketing study was conducted to conform with the Medical Device Regulation (MDR) and met its objective of gaining factual clinical evidence on the performance and safety of these marketed devices. This was further supported by the high level of patient satisfaction with the devices (> 81%) and with the speed of symptom alleviation (> 76%).

A requirement for over-the-counter medical devices is that the safety margin is such that the benefits of treatment outweigh the risks. Both products were very well tolerated with everyday use in the current study and no new side effects were identified, reflecting findings with topical use of sucralfate-containing treatments for hemorrhoidal disease as well as other indications [12, 19, 22, 31, 39, 40]. Sucralfate has a well-established safety profile without systemic action [34, 41, 42]. The topical route of administration and lack of systemic action ensure the treatments can be used without risk by patients with a range of comorbidities and receiving concomitant medications, who form a large part of the hemorrhoid population. Previous research has also shown that concomitant administration of topical sucralfate is associated with a reduction in analgesic use [38, 43].

Hemorrhoidal disease is common during pregnancy, particularly in the third trimester [44] and while this investigation did not seek to target pregnant or breastfeeding women, one of the participants was pregnant during the study period. This patient used the suppository without any incidents and benefited from symptom improvement. Pregnancy and breastfeeding pose a significant challenge to clinicians searching for effective medications whose ingredients are not harmful to the fetus or the breastfed newborn. However, while pregnancy and breastfeeding are not a contraindication to the use of sucralfate-based products, they should only be used under medical supervision during this period.

### Study limitations and strengths

This study was limited by its small sample size and lack of a comparator arm. Nevertheless, the results verified the effectiveness of the medical devices for their intended therapeutic use under real-life conditions and confirmed that they can be safely used in the comfort of the patients’ home. The topical treatments can be used by patients taking a range of concomitant medications, and their availability as an ointment or suppository addresses different patient preferences for hemorrhoid treatment.

## Conclusions

The results of this Hungarian prospective, observational study show that the sucralfate-containing rectal products, either as a suppository or ointment, are viable options for the topical treatment and management of hemorrhoidal symptoms and are safe for use in everyday settings. Furthermore, as they achieve their symptom-relieving effects by physical means, there is a reduced risk of inadvertent drug interactions. Controlled studies would be useful to further confirm the results and to assess their benefit in specific subgroups. Overall, the use of sucralfate-containing medical devices represents a promising treatment option for individuals with hemorrhoidal symptoms.

## Data Availability

Data are available from the corresponding author upon reasonable request.

## References

[1] Ganz RA (2013) The evaluation and treatment of hemorrhoids: a guide for the gastroenterologist. Clin Gastroenterol Hepatol 11:593–603. 10.1016/j.cgh.2012.12.020 23333220 10.1016/j.cgh.2012.12.020

[2] Baker H (2006) Hemorrhoids. In: Longe JL (ed) Gale encyclopedia of medicine, 3rd edn. Gale, Detroit, pp 1766–1769

[3] Sheikh P, Régnier C, Goron F, Salmat G (2020) The prevalence, characteristics and treatment of hemorrhoidal disease: results of an international web-based survey. J Comp Eff Res 9(17):1219–1232 33079605 10.2217/cer-2020-0159

[4] LeClere FB, Moss AJ, Everhart JE, Roth HP (1992) Prevalence of major digestive disorders and bowel symptoms, 1989. Adv Data. (212). pp 1–15. PMID: 10119851 10119851

[5] Riss S, Weiser FA, Schwameis K et al (2012) The prevalence of hemorrhoids in adults. Int J Colorectal Dis 27:215–220 21932016 10.1007/s00384-011-1316-3

[6] Magyar A, Csatár É (2011) A bélműködés zavarai. Székrekedés, hasmenés, aranyér. SpringMed, pp 124–125

[7] Kaidar-Person O, Person B, Wexner SD (2007) Hemorrhoidal disease: a comprehensive review. J Am Coll Surg 204(1):102–117 17189119 10.1016/j.jamcollsurg.2006.08.022

[8] De Marco S, Tiso D (2021) Lifestyle and risk factors in hemorrhoidal disease. Front Surg 8:729166. 10.3389/fsurg.2021.729166 34485376 10.3389/fsurg.2021.729166 PMC8416428

[9] Lohsiriwat V (2012) Hemorrhoids: from basic pathophysiology to clinical management. World J Gastroenterol 18:2009–2017. 10.3748/wjg.v18.i17.2009 22563187 10.3748/wjg.v18.i17.2009 PMC3342598

[10] Godeberge P, Sheikh P, Zagriadskiĭ E et al (2020) Hemorrhoidal disease and chronic venous insufficiency: Concomitance or coincidence; results of the CHORUS study (chronic venous and HemORrhoidal diseases evalUation and scientific research). J Gastroenterol Hepatol 35(4):577–585 31512275 10.1111/jgh.14857 PMC7187474

[11] Ratto C, Orefice R, Tiso D, Martinisi GB, Pietroletti R (2020) Management of hemorrhoidal disease: new generation of oral and topical treatments. Eur Rev Med Pharmacol Sci 24(18):9645–9649. 10.26355/eurrev_202009_23053 33015808 10.26355/eurrev_202009_23053

[12] Giua C, Minerba L, Piras A, Floris N, Romano F, Sifac G (2021) The effect of sucralfate-containing ointment on quality of life in people with symptoms associated with haemorrhoidal disease and its complications: the results of the EMOCARE survey. Acta Biomed 92(1):e2021029. 10.23750/abm.v92i1.11309 33683221 10.23750/abm.v92i1.11309 PMC7975930

[13] Perry KR (2019) Hemorrhoids: background, anatomy, etiology and pathophysiology. Medscape. 11-18-18:03

[14] Sebészeti_Szakmai_Kollégium (2008) Az Egészségügyi Minisztérium szakmai protokollja Aranyér betegség

[15] Wald A, Bharucha AE, Cosman BC, Whitehead WE (2014) ACG clinical guideline: management of benign anorectal disorders. Am J Gastroenterol 109(8):1141–1157. 10.1038/ajg.2014.190 25022811 10.1038/ajg.2014.190

[16] Gallo G, Martellucci J, Sturiale A et al (2020) Consensus Statement of Italian Society of colorectal surgery (SICCR): management and treatment of hemorrhoidal disease. Tech Coloproctol 24:145–164 31993837 10.1007/s10151-020-02149-1 PMC7005095

[17] Chong PS, Bartolo DC (2008) Hemorrhoids and fissure in ano. Gastroenterol Clin North Am 37(3):627–644 18794000 10.1016/j.gtc.2008.07.001

[18] Nagashima R (1981) Mechanisms of action of sucralfate. J Clin Gastroenterol 3(Suppl 2):117–127 6798100

[19] Amaturo A, Meucci M, Mari FS (2020) Treatment of haemorrhoidal disease with micronized purified flavonoid fraction and sucralfate ointment. Acta Biomed Atenei Parmensis 91(1):139–141 10.23750/abm.v91i1.9361 PMC7569574 32191669

[20] Alkhateep Y, Fareed A (2017) Double blinded randomized placebo-controlled comparative study between sucralfate ointment and lidocaine ointment after Milligan Morgan hemorrhoidectomy. Int Surg J 4(12):5

[21] Albatanony A (2016) Sucralfate ointment reduces pain and improves healing following haemorrhoidectomy: a prospective, randomized, controlled and double-blinded study. Egypt J Surg 35(2):102–105

[22] Gupta PJ, Heda PS, Kalaskar S, Tamaskar VP (2008) Topical sucralfate decreases pain after hemorrhoidectomy and improves healing: a randomized, blinded, controlled study. Dis Colon Rectum 51(2):231–234 18095028 10.1007/s10350-007-9092-4

[23] Thaha MA, Campbell KL, Kazmi SA et al (2009) Prospective randomised multi-centre trial comparing the clinical efficacy, safety and patient acceptability of circular stapled anopexy with closed diathermy haemorrhoidectomy. Gut 58(5):668–678 19091821 10.1136/gut.2008.151266

[24] Goligher JC (1980) Surgery of the anus, rectum and colon, 4th edn. Ballierè Tindal, London

[25] Dekker L, Han-Geurts IJM, Grossi U, Gallo G, Veldkamp R (2022) Is the Goligher classification a valid tool in clinical practice and research for hemorrhoidal disease? Tech Coloproctol 26(5):387–392 35141793 10.1007/s10151-022-02591-3 PMC9018630

[26] Pearson MJ, Smart NA (2018) Reported methods for handling missing change standard deviations in meta-analyses of exercise therapy interventions in patients with heart failure: a systematic review. PLoS ONE 13(10):e0205952 30335861 10.1371/journal.pone.0205952 PMC6193694

[27] Bell ML, Fiero M, Horton NJ, Hsu CH (2014) Handling missing data in RCTs: a review of the top medical journals. BMC Med Res Methodol 14:1–8 25407057 10.1186/1471-2288-14-118 PMC4247714

[28] Little RJ, D’Agostino R, Cohen ML et al (2012) The prevention and treatment of missing data in clinical trials. New Engl J Med 367(14):1355–1360 23034025 10.1056/NEJMsr1203730 PMC3771340

[29] Rørvik HD, Davidsen M, Gierløff MC, Brandstrup B, Olaison G (2023) Quality of life in patients with hemorrhoidal disease. Surg Open Sci 12:22–28 36876020 10.1016/j.sopen.2023.02.004 PMC9978033

[30] Hollander D, Tarnawski A (1990) The protective and therapeutic mechanisms of sucralfate. Scand J Gastroenterol Suppl 173:1–5 2190304 10.3109/00365529009091917

[31] Masuelli L, Tumino G, Turriziani M, Modesti A, Bei R (2010) Topical use of sucralfate in epithelial wound healing: clinical evidences and molecular mechanisms of action. Recent Pat Inflamm Allergy Drug Discov 4:25–36 19832693 10.2174/187221310789895649

[32] Koshariya M, Shitole A, Agarwal V, Dave S (2018) Role of topical sucralfate in healing of burn wounds. Int Surg J 5(9):2995

[33] Markham T, Kennedy F, Collins P (2000) Topical sucralfate for erosive irritant diaper dermatitis. Arch Dermatol 136(10):1199–1200 11030763 10.1001/archderm.136.10.1199

[34] McCullough RW (2021) Barrier therapies supporting the biology of the mucosal barrier-medical devices for common clinical mucosal disorders. Transl Gastroenterol Hepatol 6:15 33409409 10.21037/tgh.2020.02.02 PMC7724181

[35] Albahri G, Badran A, Hijazi A et al (2023) The therapeutic wound healing bioactivities of various medicinal plants. Life (Basel) 13(2):317 36836674 10.3390/life13020317 PMC9960863

[36] Piazza S, Martinelli G, Vrhovsek U et al (2022) Anti-inflammatory and anti-acne effects of *Hamamelis virginiana* bark in human keratinocytes. Antioxid (Basel) 11(6):1119 10.3390/antiox11061119 PMC9220085 35740016

[37] Morgado PJ, Suárez JA, Gómez LG, Morgado PJ (1988) Histoclinical basis for a new classification of hemorrhoidal disease. Dis Colon Rectum 31:474–480 3378471 10.1007/BF02552621

[38] Vejdan AK, Khosravi M, Amirian Z et al (2020) Evaluation of the efficacy of topical sucralfate on healing haemorrhoidectomy incision wounds and reducing pain severity: a randomised clinical trial. Int Wound J 17(4):1047–1051 32319175 10.1111/iwj.13369 PMC7948644

[39] Gupta PJ, Heda PS, Shrirao SA, Kalaskar SS (2011) Topical sucralfate treatment of anal fistulotomy wounds: a randomized placebo-controlled trial. Dis Colon Rectum 54(6):699–704 21552054 10.1007/DCR.0b013e31820fcd89

[40] Tumino G, Masuelli L, Bei R, Simonelli L, Santoro A, Francipane S (2008) Topical treatment of chronic venous ulcers with sucralfate: a placebo controlled randomized study. Int J Mol Med 22(1):17–23 18575771

[41] Rudiman R, Hanafi RV, Evan C, Halim F (2023) The efficacy of topical sucralfate in improving pain and wound healing after haemorrhoidectomy procedure: a systematic review, meta-analysis, and meta-regression of randomised clinical trials. Int Wound J 20(2):543–553 35864080 10.1111/iwj.13901 PMC9885481

[42] Kudaravalli P, John S (2023) Sucralfate. [Updated 2022 Feb 25]. StatPearls [Internet]. StatPearls Publishing, Treasure Island (FL)

[43] Ala S, Saeedi M, Eshghi F, Rafati M, Hejazi V, Hadianamrei R (2013) Efficacy of 10% sucralfate ointment in the reduction of acute postoperative pain after open hemorrhoidectomy: a prospective, double-blind, randomized, placebo-controlled trial. World J Surg 37(1):233–238 23010700 10.1007/s00268-012-1805-8

[44] Staroselsky A, Nava-Ocampo AA, Vohra S, Koren G (2008) Hemorrhoids in pregnancy. Can Fam Physician 54(2):189–190 18272631 PMC2278306

